# Assessment of Physicochemical Quality, Antioxidant Content and Activity, and Inhibition of Cholinesterase between Unripe and Ripe Blueberry Fruit

**DOI:** 10.3390/foods9060690

**Published:** 2020-05-26

**Authors:** Hyesung Hwang, Young-Jun Kim, Youngjae Shin

**Affiliations:** 1 Department of Environmental Horticulture, Dankook University, Cheonan, Chungnam 31116, Korea; sug7764@hanmail.net; 2 Department of Food Science and Technology, Seoul National University of Science and Technology, Seoul 01811, Korea; 3 Department of Food Engineering, Dankook University, Cheonan, Chungnam 31116, Korea

**Keywords:** blueberry, cultivar, maturity, antioxidant, acetylcholinesterase, butyrylcholinesterase

## Abstract

Five Korean blueberries (’’Nelson’’, ’’Duke ’’, ’’Bluejay ’’, ’’Toro’’, and ’’Elliot ’’) were harvested at two maturity stages (unripe and ripe) to evaluate fruit quality and antioxidant activities. The Hunter L, a, and b color of ripe blueberries was lower than that of unripe fruit. Soluble solid concentration (SSC) and pH increased, and titratable acidity (TA) and firmness decreased as the blueberries matured. The ripe blueberry fruits showed a higher SSC/TA ratio than the unripe fruits. Although total anthocyanin, flavonoids, phenolics content, and antioxidant activity were higher in ripe blueberries than in unripe fruit, the unripe fruit had higher acetylcholinesterase (AChE) and butyrylcholinesterase (BChE) inhibition activities than ripe fruit in all cultivars. Total antioxidant activity was highly correlated with total flavonoids and phenolics. The relationships between the total antioxidant activity and the AChE or BChE inhibitory activity are negative. There were several physicochemical quality and antioxidant activity differences in blueberries, depending on the cultivar and the maturity at harvest. Unripe fruits also contain potential health-promoting bioactive compounds as functional food ingredients.

## 1. Introduction

Fruits and vegetables contain many different phytochemicals [1,2]. Many studies have reported the higher intake of fruits and vegetables can reduce the risk of developing chronic disease [3,4]. Berries contain many bioactive compounds and are reported to have high antioxidant [5], antitumor [6,7] and anti-inflammatory activities [8]. Among the various berries such as blueberries, strawberries, blackberries, and cranberries are consumed not only in raw form but also in the form of beverages, yoghurt, jelly, and jam as processed foods. In many in vitro and in vivo experiments, berries are known to reduce the risk of cancer because they contain abundant amounts of phenolic acid, flavonoids, tannins, stilbenes, vitamin C, and vitamin E [6,7,9].

Blueberries (*Vaccinium* spp.) are classified into three types, namely the low-bush, high-bush, and rabbit-eye blueberries [10]. Low-bush blueberries are wild blueberries native to the northeast of the United States and Canada. These blueberries grow to about 15–30 cm in height; the fruit are harvested between July and September and are mainly used as raw fruit or are processed as frozen or canned fruit. High-bush blueberries are mainly grown in Florida and southern Michigan in the US as well as in eastern Canada. Their average height is about 2–3 m. Rabbit-eye blueberries are native to southeastern US and are grown in warmer winters as they have less cold hardiness [10,11].

Studies comparing the physicochemical qualities and antioxidant activities of blueberries have been conducted according to cultivar, harvest maturity, and storage period [12,13,14]. In Korea, the chemical composition of blueberries and the analysis of antioxidant contents and activities have also been reported [11,15]. Antioxidants inhibit the lipid oxidation induced by free radicals in the human body and are highly regarded as physiologically active substances that prevent carcinogenesis and aging by preventing chronic diseases, such as hypertension and diabetes. They also reduce the levels of reactive oxygen species (ROS) by preventing inflammation, cancer, and aging [9]. According to Singh et al. [16], ROS cause neurodegenerative diseases in the human brain. Dementia can be classified as cerebrovascular dementia and Alzheimer’s type dementia. Alzheimer’s disease (AD) is known to result from a decrease in the levels of the neurotransmitter, acetylcholine [17]. Despite numerous research efforts, the cause of AD is not fully understood. Acetylcholinesterase (AChE) is a key enzyme that hydrolyses acetylcholine to choline and acetic acid. Therefore, the inhibition of AChE has emerged as a commonly used treatment against AD. Treatment via the inhibition of AChE and butyrylcholinesterase (BChE) temporarily increases the levels of acetylcholine, which is important for memory [18].

The antioxidative effects of polyphenols in plants are expected to have a potential effect on the improvement of memory loss in AD [17,18,19]. In addition, studies on the cholinesterase inhibitory activity of fruits and vegetables grown in Korea are insufficient, and there is a lack of data of comparative analysis of the antioxidants in blueberries according to the cultivar and maturity stage at harvest. Therefore, the objective of this study was to compare the antioxidant contents, activities, and cholinesterase inhibitory activity according to the maturity stage and cultivar of blueberries.

## 2. Materials and Methods

### 2.1. Experimental Materials and Reagents

The blueberries used in the present study consisted of five cultivars, “Nelson”, “Toro”, “Duke”, “Bluejay”, and “Elliot” from a farm in Cheonan, Chungnam-Province. Both the unripe (50% of fruit surface turns into purple color) and ripe (100% of fruit surface turns into dark purple color) fruits were harvested. All fruit used in the experiment had a uniform size. Folin-Ciocalteu’s phenol reagent, 2,2-diphenyl-1-picrylhydrazyl (DPPH), 2,2-azino-bis(3-ethylbenzothiazoline-6-sulfonic acid) (ABTS), sodium nitrite, aluminum chloride hexahydrate, (+)-catechin, gallic acid monohydrate reagent, potassium chloride, sodium acetate anhydrous, hydrochloric acid, and sodium carbonate anhydrous were purchased from Sigma (St. Louis, MO, USA). Other reagents used were of analytical grade.

### 2.2. Evaluation of Blueberry Physicochemical Qualities

The Hunter Lab values were measured using a color meter (Chroma Meter CR-400, Minolta, Tokyo, Japan) to assess fruit color change. The color of the fruits was represented as L value (lightness ranging from 0 = black to 100 = white), a value (redness), and b value (yellowness). Thirty berries from each cultivar were measured, and the mean value was calculated. The readings were taken around the equatorial region of each berry. Fruit firmness was measured using a fruit hardness tester (FHM-1, Takemura Co., Tokyo, Japan), where the maximum resistance was measured at the moment when the 5.0-mm diameter probe penetrated the fruit from the side; it is expressed in Newton (N). The soluble solids content (SSC) of the fruit was measured by grinding the whole fruit in a commercial blender (HR20011, Phillips, Carson, NV, USA). The SSC was measured three times using a digital glucose refractometer (PAL-1, Atago, Tokyo, Japan) [5]. Titratable acidity (TA) was determined by mixing 2 g of the ground sample with 200 mL of distilled water and performing a neutralization titration with 0.1 N NaOH and 2–4 drops of 1% phenolphthalein solution. The pH was measured using a pH meter (Starter300, Ohaus Co., Ltd., Parsippany, NJ, USA).

### 2.3. Blueberry Organic Acid Content

Organic acids were analyzed using the method by Kim and Shin [20] with some modifications. For individual organic acid analysis, the Agilent 1100 Series with a diode array detector was used. The extract was diluted 10-fold with distilled water, and the samples were filtered through a 0.2-μm syringe. A Prevail organic acid column (250 × 4.6 mm id., 5 μm, Alltech, Deerfield, IL, USA) was used at 25 °C. The high-performance liquid chromatography (HPLC) mobile phase was 25 mM KH_2_PO_4_ adjusted to pH 2.1 using H_3_PO_4_, and the diode array detector was positioned at 210 nm with a 1.0 mL/min flow rate. An injection volume of 10 μL was used for the analysis. For the calibration curve, three different points (4, 20, and 100 mg/100 g) were obtained with the standard solution (oxalic acid, tartaric acid, malic acid, lactic acid, acetic acid, citric acid, succinic acid, and fumaric acid), and the results are expressed as mg/100 g of fresh weight (FW).

### 2.4. Blueberry Sugar Content

The quantification of sugars was performed using the method described by Kim and Shin [20] with some modifications. After the blueberry extract was diluted 10-fold with distilled water, the sample was filtered through a 0.45-μm syringe filter to perform HPLC analysis. Multiple HPLC analyses were performed using the Agilent 1200 series with a Refractive Index (RI) detector. For the separation of individual sugars, a carbohydrate high-performance column (250 × 4.6 mm i.d., 4 μm, Waters, Milford, MA, USA) was used at 30 °C. The mobile phase was 81% acetonitrile in distilled water at a flow rate of 1.0 mL/min. The injection amount was 10 μL. Sugar standards (glucose, fructose, sucrose, and maltose) were purchased from Sigma (St. Louis, USA). The calibration curves for three different points (200, 500, and 1000 mg/100 g) were obtained using standard solutions, and the results are expressed as mg/100 g FW.

### 2.5. Blueberry Extraction for the Measurement of Antioxidant and AChE and BChE Inhibitory Activity

The blueberries were sliced and frozen with liquid nitrogen for use in the experiment. After adding 80% ethanol to 40 g of frozen blueberries, a commercial blender (HR20011, Philips, Carson, NV, USA) was used to homogenize the berries for 3 min. The homogenized solution was filtered through a Whatman #1 paper filter, and the filtered solution was concentrated in a rotary evaporator (N-1000, Eyela, Tokyo, Japan) at 45 °C [5]. The concentrated extractions were stored at −20 °C and used for the measurement of total anthocyanin content, total flavonoid content, total phenolic content, total antioxidant activity, and AChE and BChE inhibitory activities.

### 2.6. Blueberry Total Anthocyanin Analysis

The pH differential method was used for total anthocyanin analysis [21]. The extracted samples were diluted 10-fold with pH 1.0 buffer and 10-fold with pH 4.5 buffer, and the absorbance at 510 and 700 nm was measured using a spectrophotometer (Optizen POP, Mecasys, Daejeon, Korea). The total anthocyanin content was calculated using the following equation and expressed as mg cyanidin 3-glucoside equivalents (CGE)/100 g FW.

Total anthocyanin content (mg CGE/100 g FW) =$\frac{\left(A\times \mathrm{MW}\times D\times 1000\right)}{\varepsilon }$

A (absorbance value) = [(A510 nm−A700 nm)_pH 1.0_ − (A510 nm−A700 nm)_pH 4.5_]

MW (cyanidin 3-glucoside molecular weight) = 449.2

D (dilution factor) = dilution factor

ε (cyanidin 3-glucoside molar extinction coefficient) = 26,900

### 2.7. Blueberry Total Flavonoid Analysis

Total flavonoid content was measured by colorimetric analysis [22,23]. To a 15-mL test tube containing 4 mL of distilled water and 1 mL of blueberry extract, we added 0.3 mL of 5% NaNO_2_. The mixture was vortexed and left for 5 min at room temperature. Then, 0.3 mL of 10% AlCl_3_ was added and the mixture was vortexed and left at room temperature for 6 min. After 2.4 mL of distilled water was added to 2 mL of 1 N NaOH, the mixture was vortexed and the final volume was adjusted to 10 mL; then, the absorbance at 510 nm was measured. Catechin was used as a standard, and the results are expressed in mg catechin equivalents (CE)/100 g FW.

### 2.8. Blueberry Total Phenolic Analysis

Total phenolic content was measured using the Folin–Ciocalteu colorimetric method [22,24]. After adding 0.2 mL of blueberry extract to a 15-mL test tube containing 2.6 mL of deionized water, 0.2 mL of Folin–Ciocalteu reagent was added and the mixture was vortexed and left at room temperature for 6 min. Then, 2 mL of 7% Na_2_CO_3_ was added and the mixture was left for 90 min in a dark room; finally, the absorbance was measured at 750 nm. Gallic acid was used as a standard, and the results are expressed in mg gallic acid equivalent (GAE)/100 g FW.

### 2.9. Blueberry DPPH Radical Scavenging Activity Analysis

The DPPH radical scavenging activity was measured using a modified method described by Mira-Sánchez et al. [25]. The DPPH solution was prepared by dissolving 0.00789 g powdered DPPH in 200 mL methanol. The solution was diluted to an optical density (OD) value of 0.65 at 517 nm using 80% methanol. After adding 2.95 mL of DPPH solution to 50 µL of the blueberry extract, the mixture was left for 30 min in a dark room; then, the absorbance was measured at 517 nm. Antioxidant activity by DPPH radical scavenging activity is expressed as mg vitamin C equivalent (VCE)/100 g FW.

### 2.10. Blueberry ABTS Radical Scavenging Activity Analysis

The ABTS radical scavenging activity of the extracted samples was measured using ABTS radicals [26]. After mixing 1 mM 2,2′-Azobis (2-amidinopropane) dihydrochloride (AAPH) with 100 mL of 1× phosphate buffer saline (PBS) solution and 2.5 mM ABTS, the mixture was allowed to react for 40 min in a 70 °C water bath. Subsequently, the PBS solution was used to dilute the mixture to an OD value of 0.65 at 734 nm. The test solution containing 20 μL of the diluted solution mixed with deionized water and 980 μL of the ABTS reaction solution was reacted at 37 °C for 10 min. Then, the absorbance was measured at 734 nm. The results are expressed in mg vitamin C equivalents (VCE)/100 g FW.

### 2.11. Blueberry AChE and BChE Inhibitory Activity Analysis

AChE inhibitory activity was measured using the Ellman colorimetric method in 96-well microplate [27]. For the enzymatic reaction, 150 µL of PBS was dispensed, followed by reaction with blueberry fruit extract (10 mg/mL) and 20 µL of 0.2 U AChE. Subsequently, 30 µL of 5,5-dithio-bis (2-nitrobenzoic acid) and 20 µL of 15 mM acetylthiocholine iodide were added and allowed to react for 30 min at 37 °C. Then, the absorbance was measured at 415 nm using a microplate reader (Versa max, Molecular Devices, Sunnyvale, CA, USA). Tacrine was used as a positive control, and the results are expressed as the inhibition rate (%). The BChE inhibitory activity was also measured using a modified Ellman’s method [27]. Tacrine was used as the positive control, and the results are also expressed as the inhibition rate (%).

Cholinesterase inhibitory activity (%) = (1 − absorbance of sample or positive control/absorbance of negative control) × 100

### 2.12. Statistical Analysis

For statistical analysis, analysis of variance (ANOVA) was performed using SAS version 9.3 (SAS Institute, Inc., Cary, NC, USA), and significant differences in the results were expressed in confidence intervals of 95% using Duncan’s multiple range test. Pearson correlations were used to quantify the relationships between parameters. The data are expressed as the mean ± standard deviation for triplicate determinations.

## 3. Results and Discussion

### 3.1. Blueberry Color

The Hunter L, a, and b color of five different blueberry cultivars at different maturity stages are shown in Table 1. The average L value of blueberries was 34.80 for the unripe fruit and 25.85 for the ripe fruit. As the fruit ripened, both the L, a, and b values decreased because the fruit color was turned to dark purple from red. Therefore, the Hunter a and b values showed a tendency to decrease from a positive value to negative in all three cultivars. The “Nelson”, “Toro”, “Duke”, “Bluejay”, and “Elliot” unripe fruit showed significantly higher Hunter b values than the values for the ripe fruit.

### 3.2. Blueberry Firmness

The average firmness of unripe fruit (6.80 N) was higher than that of ripe fruit (4.88 N) by 1.92 N. The firmness of unripe “Duke” was 7.15 ± 0.42 N and was significantly higher than that of the other cultivars. The ripe “Nelson” was 4.25 ± 0.79, the lowest compared to the other cultivars (Table 2). Shin et al. [5] also found that unripe strawberries are about twice as hard as the ripe fruit. The low firmness of the ripe fruit is due to the softening of the fruit, which is caused by the collapse of the cell walls of the fruit and the weakening of cell binding. It is known that various cell wall-degrading enzymes work in this process [28].

### 3.3. Blueberry SSC, TA, and pH

SSC and TA are important sensory factors that affect the taste of fruits. The average SSC of unripe and ripe fruit was 9.23 °Bx and 11.53 °Bx, respectively. Overall, ripe fruit showed a higher SSC than unripe fruit (Table 2). The SSC of ripe “Bluejay” and “Nelson” cultivars were 12.87 ± 0.15 °Bx and 12.67 ± 0.15 °Bx, respectively, which were significantly higher than that of the other cultivars. The SSC of unripe “Duke” showed the lowest SSC at 7.93 ± 0.25 °Bx. Unripe fruit showed a higher TA than ripe fruit. The TA of unripe “Nelson” and “Bluejay” were 2.20% ± 0.03% and 2.39% ± 0.03%, whereas the TA of ripe “Duke” was 0.46% ± 0.01%, which was the lowest among the samples (Table 2). For pH, ripe fruit had a higher pH than unripe fruit, with an average of 3.12 and 2.64, respectively (Table 2).

### 3.4. Blueberry Organic Acid Content

**Figure 1:**
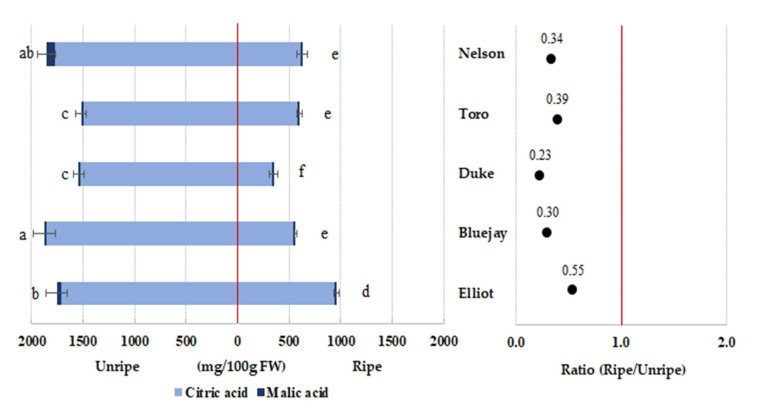
Different letters (**a**–**f**) are significant differences by Duncan’s multiple range test (*p* < 0.05).

In contrast to SSC, the TA consistently decreased as the blueberries matured (Table 2). Analysis of organic acids showed that citric acid was dominant in all cultivars (Figure 1). Total organic acid content was significantly higher in unripe than ripe blueberry. The organic acid content is highly associated with the acidity of blueberries and is also closely related to their taste. The citric acid and malic acid content decreased as the blueberries matured in all cultivars (Figure 1). Previous studies showed that the main organic acid in blueberry was citric acid, which makes up 77%–87% of the total acid, depending on the maturity stage at harvest. In addition, small amounts of succinic, tartaric, and shikimic acids were found [29]. A decrease in citric acid was reported in ripening blueberry fruit [29,30]. In our study, the citric acid content of the “Bluejay” cultivar was significantly higher in unripe fruit than in the other cultivars.

### 3.5. Blueberry Sugar Content

**Figure 2:**
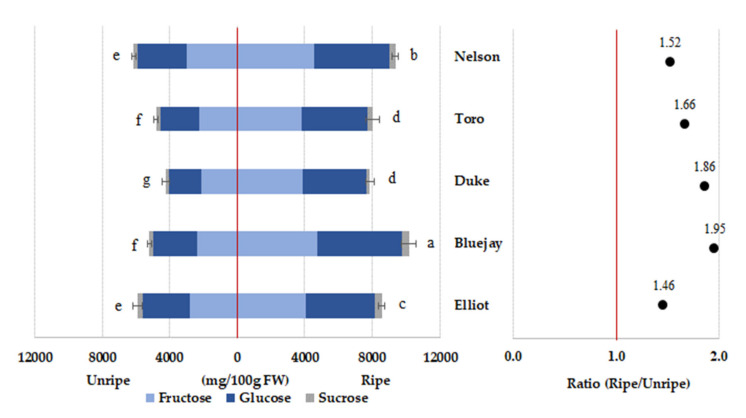
Different letters (**a**–**g**) are significant differences by Duncan’s multiple range test (*p* < 0.05).

Sugars (sucrose, glucose, and fructose) are related to the sweetness of fruit, whereas organic acids (citrate and malate) determine fruit acidity. The quantification of individual sugars (fructose, glucose, and sucrose) was analyzed in the different cultivars and maturities. Fructose and glucose were the main sugars in all blueberry cultivars, and sugar content increased as blueberries matured in all cultivars. The total sugar content of the “Bluejay” and “Nelson” cultivars at the ripe stage was significantly higher (10.18 and 9.36 g/100 g FW, respectively) than that of the other cultivars (Figure 2). Li et al. [30] reported that the three major sugars of high-bush blueberries are sucrose, fructose, and glucose. The latter two contained similar amounts and were predominant in blueberry fruit, whereas sucrose was present in relatively low concentrations; these two sugars increased during fruit maturation, which is in agreement with our results.

### 3.6. Blueberry Total Anthocyanin Content

The total blueberry fruit anthocyanin content according to cultivar and maturity was significantly higher at the ripe stage than that at the unripe stage in all five cultivars (Table 3). In this study, the blueberry fruit anthocyanin content increased with maturation. Similar findings are also reported by Siriwoharn et al. [31] that the total blackberry anthocyanin of “Marion” and “Evergreen” increases as maturation progresses. Connor et al. [13] reported that the total anthocyanin content in matured “Elliot” was 191–239 mg/100 g FW and Bunea et al. [14] reported that the anthocyanin content in “Elliot” was 163.4 mg/g FW. These results were higher than our results; the harvest location and the maturity stage at harvest could affect the anthocyanin content. Castrejón et al. [12] reported that total anthocyanin increases as blueberry matures and concludes that anthocyanin biosynthesis is highly related to the developmental stages of the fruit, and enzyme activities are controlled in response to different developmental and environmental cues.

### 3.7. Blueberry Total Flavonoid Content

The average total flavonoid content of unripe and ripe blueberry fruit was 57.19 and 77.14 mg/100 g FW, respectively. The total flavonoid content of the unripe and ripe “Nelson” cultivar was 79.83 and 102.66 mg/100 g FW, respectively, which was significantly higher than that of the other cultivars. The total flavonoid content of unripe “Bluejay” and ‘Elliot’ was 38.89 mg/100 g FW and 42.56 mg/100 g FW, respectively, which was significantly lower than that of the other cultivars (Table 3). Studies on the total flavonoid content according to the maturity of berry fruit have been reported. Shin et al. [5] also reported that the flavonoid content is higher in unripe strawberries than in ripe strawberries. Conversely, Hwang et al. [32] reported that the Seolhyang and Maehyang strawberry cultivars showed no significant differences between the unripe and ripe fruit, except for the Janghee cultivar. According to the aronia study, the total flavonoid content of unripe fruit is higher than that of ripe fruit in three cultivars (“Viking”, “McKenzie”, and “Kingstar K1”) [23]. These changes in the flavonoid content in berry fruit, according to maturity, may be due to changes in the content of individual flavonoid compounds, such as quercetin and kaempferol, during ripening [33].

### 3.8. Blueberry Total Phenolic Content

The average phenolic content of unripe and ripe blueberry fruit was 196.49 and 255.12 mg/100 g FW, respectively. The total blueberry phenolic content increased during the ripening process, which was a similar pattern to that of the flavonoid content in this study. Wang et al. [4] reported that the ratio of the total flavonoids to the total phenolics of 14 blueberry cultivars ranges between 18.2% and 34.2%. In our research, the ratio was 22.8% to 34.9%, which was similar to their results. The total phenolic content of the “Nelson” and “Toro” cultivars was 300.90 and 287.33 mg/100 g FW in ripe fruit and 228.51 and 227.66 mg/100 g FW in unripe fruit, respectively, which was significantly higher than the other cultivars (Table 3). In the comparison among cultivars according to maturity, the phenolic content was higher at the ripe stage in the order of “Nelson”, “Toro”, “Duke”, “Bluejay”, and “Elliot”. Connor et al. [13] also reported that total phenolic content in blueberry increases as maturation progresses, and similar results were also reported for Rubus coreanus Miquel and the red raspberry [34,35]. Conversely, Shin et al. [5] reported that unripe “Jewel” strawberries show a higher total phenolic content than ripe fruit, at 300–350 mg (GAE)/100 g FW and 230–280 mg (GAE)/100 g FW, respectively. However, Siriwoharn et al. [31] reported that blackberry phenolic content shows a gradual increase in the “Marion” cultivar as the fruit matures, whereas the “Evergreen” cultivar phenolic content decreases when maturing from the unripe to the half-ripe stage and increases at full ripeness. Thus, the phenolic content in unripe and ripe berry fruit varies according to cultivars and maturity stages.

### 3.9. Blueberry Total Antioxidant Activity

The total antioxidant activities of DPPH and ABTS radical scavenging activity of ripe blueberry were higher than that of unripe fruit, which was a similar pattern to total flavonoids and total phenolics. DPPH radical scavenging activity of ripe “Nelson” and “Toro” cultivars was 427.39 and 385.39 mg/100 g FW, respectively (Table 3). ABTS radical scavenging activity was similar to DPPH radical scavenging activity; ripe “Nelson” showed a value of 1424.10 mg/100 g FW, which was significantly higher than that of other cultivars. Among the unripe fruit, “Nelson” and “Toro” had total antioxidant activities of 975.12 and 988.75 mg/100 g FW, respectively, which was significantly higher than that of the other cultivars (Table 3). Bunea et al. [14] reported that ABTS and DPPH methods are considered to be the most appropriate to measure the antioxidant activity of blueberry fruit, in good agreement with the concentrations of phenolic derivatives (anthocyanins, flavonoids, and polyphenols). Shin et al. [5] reported that Jewel strawberries that are unripe have higher antioxidant activity than fully ripe strawberries. Hwang et al. [32] reported that the total antioxidant activity of ripe and unripe Seolhyang strawberry fruit showed no differences, whereas unripe Janghee fruit showed significantly higher activity than ripe fruit. Yang et al. [23] also reported that antioxidant compounds and activities of aronia fruit showed significantly higher values for the unripe stage than the ripe stage. Based on these results, antioxidant activity varies according to cultivar and maturity.

### 3.10. Blueberry AChE and BChE Inhibitory Activity Analysis

**Figure 3:**
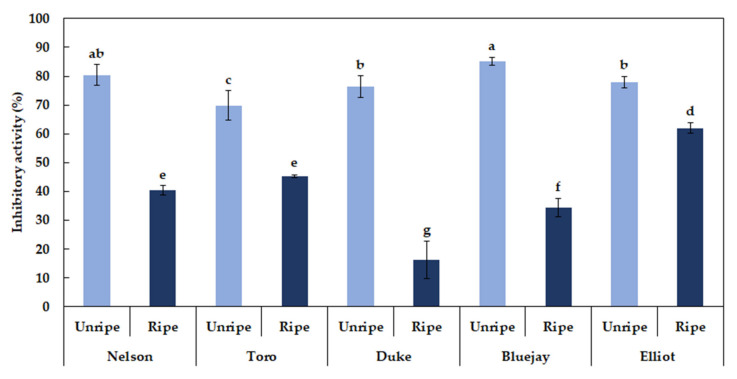
Different letters (**a**–**g**) are significant differences by Duncan’s multiple range test (*p* < 0.05).

**Figure 4:**
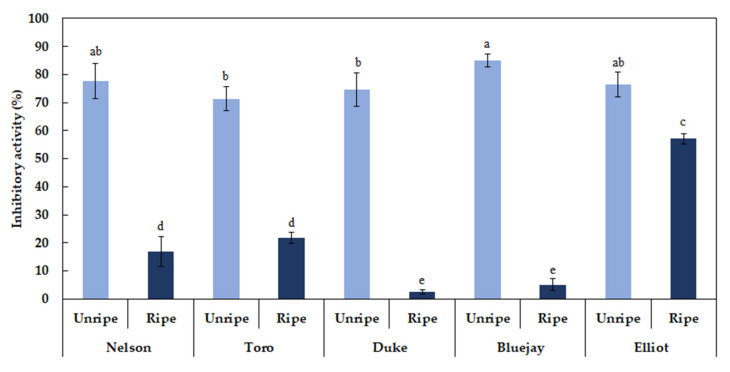
Different letters (**a**–**e**) are significant differences by Duncan’s multiple range test (*p* < 0.05).

A decrease of acetylcholine causes AD, and AChE is the major enzyme that suppresses acetylcholine levels [17,18,36]. The average AChE inhibitory activity of unripe and ripe blueberry extracts (1 mg/mL) was 77.97% and 39.69%, respectively. The inhibitory activity was higher at the unripe stage in the order of “Bluejay” (85.19%), “Nelson” (80.47%), “Elliot” (77.95%), “Duke” (76.42%), and “Toro” (69.84%) (Figure 3). The average BChE inhibitory activity of unripe and ripe blueberry extracts (1 mg/mL) was 77.09% and 20.68%, respectively. The average inhibitory activity rate (%) of unripe fruit was approximately 3.7 times higher than that of ripe fruit (Figure 4). Currently, only a few studies on the AChE inhibitory activity of berry fruit have been reported. Hwang et al. [32] reported that unripe strawberries showed a significantly higher AChE inhibition rate than the ripe fruit. A study on the AChE inhibition activity has also been attempted in medicinal plants. According to Jung et al. [19], *Schisandra chinensis*, *Hovenia dulcis*, *Thuja orientalis*, and *Eleutherococcus senticosus* showed relatively high AChE inhibitory activities at 1 mg/mL final concentration, which were 33.0%, 26.6%, 20.7%, and 17.8%, respectively. Extracts of *Angelica gigas*, *Polygala tenuifolia*, *Cnidium officinale*, *Poria cocos*, and *Acorus gramineus* showed approximately 6%–10% inhibitory activity.

### 3.11. Blueberry Correlation between Blueberry Antioxidant Content and Activity

Pearson correlations were used to determine the relationship between antioxidant compounds and activity (Table 4). Among the blueberry cultivars, the total phenolic content was highly correlated to the DPPH and ABTS radical scavenging activity by R = 0.974 and 0.964, respectively. The total blueberry phenolic content was also strongly correlated with total flavonoid content (R = 0.903). Bunea et al. [14] reported that the ferric reducing antioxidant power (FRAP), ABTS, and DPPH assays are highly significantly correlated; moreover, total polyphenol content and total anthocyanin content in blueberries also showed strong correlations. Further, Shin et al. [5] reported that the total antioxidant activity of strawberry is highly correlated with total flavonoids and phenolics and that the relationships between total phenolics and flavonoids are also strong, which agree with the results of the present study. However, the relationship between total antioxidant activities measured using the DPPH and ABTS methods was strong (R = 0.956); the total flavonoid content was highly correlated with DPPH and ABTS radical scavenging activity, R = 0.882 and 0.857, respectively, but not with the AChE (R = −0.542) or BChE (R = −0.597) inhibitory activities. Several studies have reported that the total phenolic concentrations of fruits are positively related to the total antioxidant activity [4,5,23,32]. The relationships between the total antioxidant activity and the AChE or BChE inhibitory activity are negative in this study because the unripe fruits showed higher cholinesterase inhibitory activity than ripe fruits.

## 4. Conclusions

In the present study, physicochemical properties, antioxidant compounds, antioxidant activities, and AChE and BChE inhibition activities of five blueberry cultivars harvested at different ripening stages were investigated. The results showed that total phenolic, flavonoid, anthocyanin content, and antioxidant activity were higher in ripe blueberries than in unripe fruit. On the contrary, the AChE and BChE inhibitory activities were higher at the unripe stage. This study is highly significant because only a few studies on the AChE inhibitory activity of berry fruit have been reported so far. Among the blueberry cultivars, the total phenolic content was highly correlated to the DPPH and ABTS radical scavenging activity. The total blueberry phenolic content was also strongly correlated with total flavonoid content. Although the ripe blueberry fruits contain comparatively more abundant antioxidative effects and higher SSC/TA ratios than the unripe fruits, the unripe blueberries show a high cholinesterase inhibitory activity, and they could use as a potential promising ingredient for developing functional foods.

## Figures and Tables

**Table 1:** Hunter L, a, b color of blueberry fruits at different maturity stages.

Cultivars	Maturity Stages	Hunter L (Lightness)	Hunter a (Redness)	Hunter b (Yellowness)
Nelson	Unripe	36.19 ± 5.12 ^b^	7.65 ± 3.04 ^bc^	3.11 ± 3.69 ^b^
	Ripe	24.15 ± 1.45 ^f^	−0.04 ± 0.34 ^d^	−2.04 ± 0.80 ^d^
Toro	Unripe	33.43 ± 2.89 ^c^	10.84 ± 2.62 ^a^	2.29 ± 2.21 ^bc^
	Ripe	26.30 ± 1.05 ^de^	−0.21 ± 0.14 ^d^	−3.08 ± 0.44 ^de^
Duke	Unripe	34.11 ± 2.95 ^c^	8.31 ± 2.39 ^b^	2.42 ± 2.46 ^bc^
	Ripe	26.17 ± 1.03 ^de^	−0.25 ± 0.08 ^d^	−2.89 ± 0.65 ^de^
Bluejay	Unripe	37.68 ± 4.67 ^a^	7.01 ± 3.82 ^c^	6.01 ± 2.71 ^a^
	Ripe	27.15 ± 1.65 ^d^	−0.13 ± 0.21 ^d^	−3.38 ± 0.60 ^e^
Elliot	Unripe	32.60 ± 3.33 ^c^	8.46 ± 2.22 ^b^	1.78 ± 2.06 ^c^
	Ripe	25.46 ± 1.24 ^ef^	0.17 ± 0.33 ^d^	−2.75 ± 0.70 ^de^

Date are means ± standard deviation. ^a–f^ Values in the same column not sharing a common superscript are significantly different by Duncan’s multiple range test (*p* < 0.05).

**Table 2:** Firmness, Soluble solid content, titratable acidity, and pH of blueberry fruits at different maturity stages.

Cultivars	Maturity Stages	Firmness (N/5 mmØ)	Soluble Solid Concentration (°Brix)	Titratable Acidity (%)	SSC/TA Ratio	pH
Nelson	Unripe Ripe	6.71 ± 0.69 ^bc^ 4.25 ± 0.79 ^f^	10.23 ± 0.06 ^c^ 12.67 ± 0.15 ^a^	2.20 ± 0.03 ^b^ 0.77 ± 0.02 ^f^	4.65 16.45	2.66 ± 0.01 ^f^ 3.12 ± 0.02 ^c^
Toro	Unripe Ripe	6.87 ± 0.57 ^ab^ 5.38 ± 0.54 ^d^	8.53 ± 0.15 ^f^ 10.30 ± 0.10 ^c^	2.08 ± 0.02 ^c^ 0.64 ± 0.01^g^	4.10 16.09	2.62± 0.01 ^fg^ 3.07 ± 0.04 ^d^
Duke	Unripe Ripe	7.15 ± 0.42 ^a^ 5.09 ± 0.58 ^de^	7.93 ± 0.25 ^g^ 10.27 ± 0.15 ^c^	1.90 ± 0.03 ^d^ 0.46 ± 0.01 ^h^	4.17 22.33	2.60 ± 0.01 ^g^ 3.31 ± 0.02 ^a^
Bluejay	Unripe Ripe	6.78 ± 0.51 ^bc^ 4.78 ± 0.69 ^e^	9.57 ± 0.06 ^e^ 12.87 ± 0.15 ^a^	2.39 ± 0.03 ^a^ 0.66 ± 0.01 ^g^	4.00 19.50	2.64 ± 0.02 ^fg^ 3.26 ± 0.05 ^b^
Elliot	Unripe Ripe	6.51 ± 0.82 ^c^ 4.91 ± 0.61 ^e^	9.87 ± 0.06 ^d^ 11.53 ± 0.06 ^b^	2.10 ± 0.01 ^c^ 1.07 ± 0.02 ^e^	4.70 10.78	2.66 ± 0.01 ^f^ 2.85 ± 0.04 ^e^

Date are means ± standard deviation. ^a–g^ Values in the same column not sharing a common superscript are significantly different by Duncan’s multiple range test (*p* < 0.05). SSC/TA ratio is calculated by SSC (°Brix)/titratable acidity (%).

**Table 3:** Antioxidant content and activities of blueberry fruits at different maturity stages.

Cultivars	Maturity Stages	Total Anthocyanins (mg/100 g)	Total Flavonoids (mg CE/100 g)	Total Phenolics (mg GAE/100 g)	DPPH (mg/100 g)	ABTS (mg/100 g)
Nelson	Unripe Ripe	19.67 ± 0.06 ^e^ 136.51 ± 9.95 ^ab^	79.83 ± 6.86 ^bc^ 102.66 ± 9.70 ^a^	228.51 ± 3.17 ^b^ 300.90 ± 12.99 ^a^	271.69 ± 11.67 ^bc^ 427.39 ± 26.16 ^a^	975.12 ± 41.90 ^d^ 1424.10 ± 102.54 ^a^
Toro	Unripe Ripe	40.28 ± 1.38 ^d^ 145.20 ± 10.13 ^a^	66.20 ± 4.30 ^de^ 80.94 ± 2.18 ^b^	227.66 ± 5.05 ^b^ 287.33 ± 13.18 ^a^	279.75 ± 9.72 ^bc^ 385.39 ± 26.83 ^a^	988.75 ± 57.41 ^d^ 1304.22 ± 60.77 ^b^
Duke	Unripe Ripe	28.19 ± 2.30 ^e^ 134.76 ± 8.69 ^b^	58.45 ± 2.30 ^e^ 71.78 ± 0.84 ^cd^	184.73 ± 7.67 ^d^ 242.46 ± 0.83 ^b^	183.01 ± 66.40 ^ef^ 322.17 ± 12.03 ^b^	830.32 ± 39.45 ^ef^ 1176.34 ± 76.06 ^c^
Bluejay	Unripe Ripe	21.81 ± 2.72 ^e^ 137.10 ± 0.14 ^ab^	38.89 ± 2.53 ^f^ 67.41 ± 3.80 ^d^	154.80 ± 0.36 ^e^ 241.61 ± 6.49 ^b^	151.19 ± 24.95 ^f^ 305.20 ± 1.47 ^b^	725.01 ± 61.02 ^f^ 1159.88 ± 84.77 ^c^
Elliot	Unripe Ripe	27.39 ± 2.51 ^e^ 70.89 ± 1.64 ^c^	42.56 ± 6.05 ^f^ 62.93 ± 2.12 ^de^	186.76 ± 14.67 ^d^ 203.31 ± 1.92 ^c^	202.11 ± 29.50 ^de^ 234.35 ± 2.65 ^cd^	795.06 ± 93.90 ^ef^ 874.04 ± 40.52 ^de^

Date are means ± standard deviation (mg/100 g fresh weight (FW)). ^a–g^ Values in the same column not sharing a common superscript are significantly different by Duncan’s multiple range test (*p* < 0.05).

**Table 4:** Pearson correlation (*R*) between antioxidant compounds, antioxidant activities, and AChE and BChE inhibitory activity of blueberry fruits.

	Total Anthocyanins	Total Flavonoids	Total Phenolics	DPPH	ABTS	AChE
Total flavonoids	0.621 **					
Total phenolics	0.801 **	0.903 **				
DPPH	0.806 **	0.882 **	0.974 **			
ABTS	0.859 **	0.857 **	0.964 **	0.956 **		
AChE	−0.928 **	−0.542 **	−0.685 **	−0.714 **	−0.770 **	
BChE	−0.969 **	−0.597 **	−0.748 **	−0.768 **	−0.825 **	0.972 **

Pearson correlation (*R*): **, significance at *p* < 0.01
